# Effectiveness of Rifaximin on the Outcomes of Irritable Bowel Syndrome: A Systematic Review and Meta-Analysis of Randomized Controlled Trials

**DOI:** 10.7759/cureus.44807

**Published:** 2023-09-06

**Authors:** Zarghuna Khan, Saad Khalid Khan, Ibrahim Reyaz, Hemalatha Anam, Osama Ijaz, Ilqa Attique, Zoha Shahzad, Faraz Saleem

**Affiliations:** 1 Internal Medicine, Rehman Medical Institute, Peshawar, PAK; 2 Medicine, Army Medical College, Rawalpindi, PAK; 3 Internal Medicine, Christian Medical College and Hospital, Ludhiana, IND; 4 Internal Medicine, Apollo Institute of Medical Sciences and Research, Hyderabad, IND; 5 Internal Medicine, Services Institute of Medical Sciences, Lahore, PAK; 6 Internal Medicine, Foundation University Medical College, Islamabad, PAK; 7 Internal Medicine, Fatima Jinnah Medical University, Lahore, PAK; 8 Internal Medicine, California Institute of Behavioral Neurosciences & Psychology, Fairfield, USA; 9 Internal Medicine, Akhtar Saeed Medical and Dental College, Lahore, PAK

**Keywords:** randomized controlled trials, meta-analysis, systematic review, irritable bowel syndrome, outcomes, rifaximin

## Abstract

Irritable bowel syndrome (IBS) is a prevalent gastrointestinal disorder that impacts the lives of many individuals worldwide. We conducted a systemic review and meta-analysis of randomized controlled trials (RCTs) to assess both the effectiveness of rifaximin in alleviating IBS symptoms and its potential adverse effects.

PubMed, Web of Science, Embase, the Cochrane Library, Scopus, and Google Scholar were searched from inception until August 20, 2023, for studies comparing rifaximin with placebo in the resolution of symptoms among IBS patients. Risk ratios (RRs) and their corresponding 95% confidence intervals (CIs) were derived for all the outcomes of interest. Six RCTs were pooled in this analysis. The results showed improved abdominal distension with rifaximin over the control group. Overall symptom relief at the end of the treatment period and follow-up period was also observed in the patients receiving rifaximin. However, no significant differences were found between the rifaximin group and the control group for the outcomes of abdominal pain, nausea, headache, vomiting, diarrhea, sinusitis, bronchitis, and upper respiratory tract infection. The results of our meta-analysis support the use of rifaximin in the treatment of IBS, owing to its safety and effectiveness. Future RCTs should be conducted to assess this topic of interest more extensively.

## Introduction and background

Irritable bowel syndrome (IBS) is a common gastrointestinal disorder, classically characterized by recurring abdominal pain accompanied by a change in bowel habits. To confirm a diagnosis of IBS, the absence of structural and biochemical abnormalities in bowel health is required [1]. Patients with IBS are usually classified by the Rome III criteria based on their symptoms: IBS with constipation (IBS-C), IBS with diarrhea (IBS-D), and mixed IBS with constipation and diarrhea (IBS-M) [2]. In recent years, the incidence of all subgroups of this syndrome has drastically increased, affecting nearly 3.8%-11.2% of individuals worldwide [3]. These individuals claim that IBS has a significant negative impact on their lives, and they complain of constant fear of incontinence due to relapsing diarrhea, accompanied by difficulties in working and socializing because of moderate to severe abdominal pain [4]. Moreover, because the pathophysiology of this disease is not well understood, medicine is usually prescribed to address only the predominant symptom experienced by the IBS patient, often leading to unsatisfactory results [5]. Thus, it is crucial to investigate adequate treatment options for IBS.

Existing and readily available treatment options for IBS include dietary changes, fiber supplements, lubiprostone, tricyclic antidepressants (TCAs), selective serotonin receptor inhibitors (SSRIs), antibiotics, and probiotic treatments. However, many of these may lead to adverse effects, such as bloating, nausea, abdominal pain, constipation, dizziness, and/or diarrhea [6, 7]. One antibiotic, rifaximin, has proven to be remarkably effective in treating bacterial infections of the intestines with minimal side effects. It has been approved by the United States Food and Drug Administration (FDA) for the management of traveler's diarrhea [8]. Rifaximin is a broad-spectrum antibiotic from the rifamycin family that hinders bacterial gene expression in both gram-positive and gram-negative organisms. Due to its low systemic absorption, the risk of adverse events associated with its administration is insignificant, indicating a high therapeutic index. Furthermore, its near non-absorbability results in the high bioavailability of this drug within the gastrointestinal tract. It also shows promising minimal inhibitory concentration values for many pathogenic microbes [9].

Numerous randomized controlled trials (RCTs) have been conducted to explore the potential benefit of rifaximin administration for treating IBS [8,10-14]. We performed a systematic review and meta-analysis of these trials to comprehensively determine the safety and efficacy of rifaximin in the treatment of irritable bowel syndrome.

## Review

Methods

This systematic review and meta-analysis were conducted in accordance with the guidelines in the Cochrane Handbook and the Preferred Reporting Items for Systematic Reviews and Meta-Analysis (PRISMA) [15].

Search Strategy

A systematic search was performed on PubMed, Web of Science, Embase, Cochrane Library, Scopus, and Google Scholar from their inception until August 20, 2023, for clinical trials that evaluated the safety and efficacy of rifaximin for the treatment of IBS. No restrictions were set on time or language. The following key terms and words analogous to them were used: 'rifaximin,' 'irritable bowel syndrome,' 'IBS,' and 'diarrhea,' along with the Boolean operators AND and OR.

Study Design and Population

Two reviewers independently read relevant articles to assess the eligibility of studies. A third reviewer was consulted in case of any discrepancies. The inclusion criteria included: 1) studies with IBS patients in two different experimental groups, including rifaximin and placebo; 2) randomized controlled trials (RCTs); 3) at least one of the primary or secondary outcomes were recorded. The exclusion criteria included: 1) observational studies, systematic reviews, narrative reviews, letters to the editor, and study protocols; 2) studies without English translation available. Details of the study selection process are available in Appendix A.

Data Extraction and Quality Assessment

Two independent researchers extracted the following data: last name of the first author, year of publication, location, study design, sample size, mean age, follow-up, number or percentage of male and female population, events/total for the overall relief of symptoms, vomiting, nausea, headache, abdominal distention, and abdominal pain. Based on this data, risk ratios with their corresponding 95% confidence intervals (CIs) were calculated. Quality assessment was performed by an independent researcher using the risk-of-bias tool 2 (RoB 2.0) (Appendix B) [16].

Statistical Analysis

Review Manager (RevMan) ((Computer Program), Version 5.4., The Cochrane Collaboration, 2020), was used for statistical analysis and to generate forest plots. Forest plots with a random or fixed-effects model were used to determine the pooled effect size. A fixed-effects model was used when I^2^ was less than or equal to 50%, and a random-effects model was used when I2 was more than or equal to 50%. A funnel plot was used to assess publication bias (Appendix C). A p-value < 0.05 indicated statistical significance.

Results

**Figure 1:**
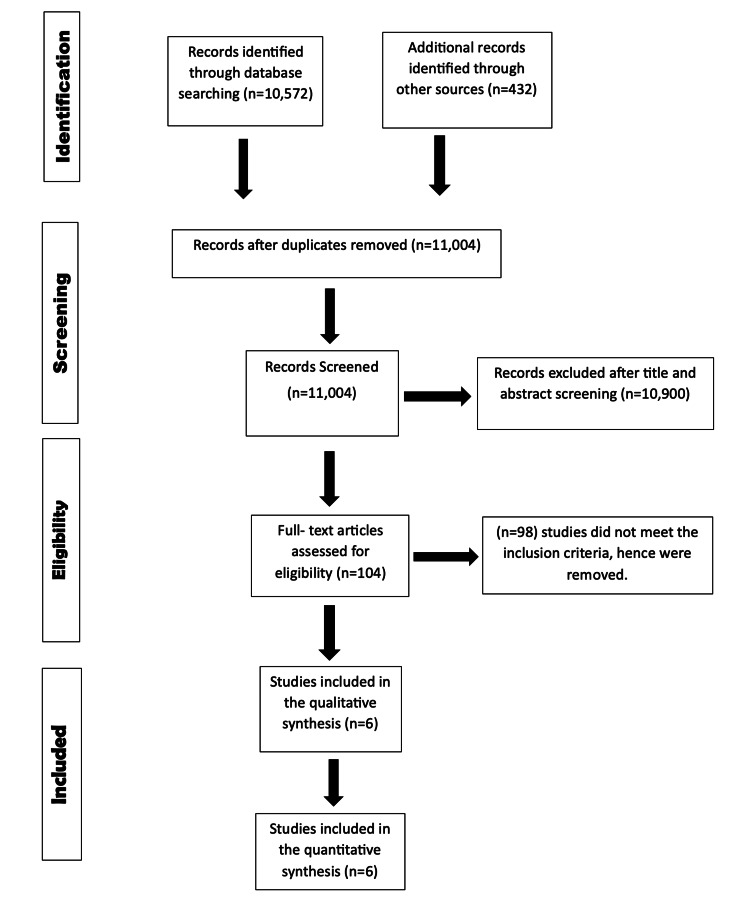
A PRISMA flow chart showcasing the process of study selection.

The PRISMA flowchart in Figure 1 illustrates a comprehensive screening process.

Initially, a total of 638 articles were retrieved. However, we included a total of six RCTs in our meta-analysis after deduplication and filtering. The baseline characteristics of the included studies are listed in Table 1.

**Table 1:** Baseline characteristics of the included studies IBS: irritable bowel syndrome; TARGET: (T-Targeted, non-systemic; A-Antibiotic; R-Rifaximin; G-Gut-selective; E-Evaluation of; T-Treatment for non-C IBS); IBS-D: IBS with diarrhea; IBS-C: IBS with constipation; IBS-M: mixed IBS with constipation and diarrhea

Author, Year	Type of study	Number of participants (experimental/control)	Mean age (years) (experimental/control)	Males (experimental/control)	Dosage	IBS type	Diagnosis criteria	Treatment duration	Follow-up
Lembo, 2008 [12]	Phase 2, double-blind, multicenter clinical trial	191/197	NA	NA	550 mg bid	IBS-D	Rome II	14 days	12 weeks
Lembo, 2016 [10]	Phase 3, randomized, double-blind, placebo-controlled trial	328/308	47.9 ± 14.2/45.6 ± 13.8	106/89	550 mg tid	IBS-D	Rome III	14 days	18 weeks
Lembo, 2020 [11]	Follow-up clinical trial of TARGET	319/302	NA	NA	550 mg tid	IBS-D	Rome III	14 days	18 weeks
Pimentel, 2006 [8]	Double-blind, randomized, placebo-controlled study	43/44	39.1 ± 12.5/ 38.2 ± 9.8	14/15	400 mg tid	NA	Rome I	10 days	10 weeks
Pimentel, 2011 [13]	Phase 3, double-blind, placebo-controlled trial (TARGET 1, TARGET 2)	309/314, 315/320	46.2 ± 15.0/ 45.5 ± 15.6, 45.9 ± 13.9/ 46.3 ± 14.6	74/92, 88/95	550 mg tid	IBS without constipation	Rome II	14 days	10 weeks
Sharara, 2006 [14]	Double-blind, randomized, placebo-controlled study	63/61 3114	42.2 ± 11.4/38.9 ± 10.6 43.58	30/26	400 mg bid	IBS-D, IBS-C, IBS-M	Rome II	10 days	10 days

The total number of patients was 3,114, with a mean age of 43.58 years. The patients had either IBS types C, D, or M. The follow-up for all the studies was more than 10 weeks, except for the study by Sharara et al. [14].

Primary outcomes

Overall Symptom Relief at the End of Treatment Period

**Figure 2:**
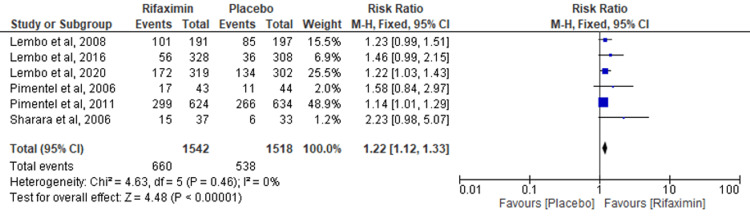
Overall symptom relief at the end of the treatment period

Six RCTs were pooled to evaluate the overall symptom relief at the end of the treatment period. The results were significant, favoring the rifaximin group over the placebo group (risk ratio (RR) = 1.22; 95% CI: 1.12-1.33; p < 0.00001; I² = 0%) (Figure 2).

Overall Symptom Relief at the End of the Follow-up Period

**Figure 3:**
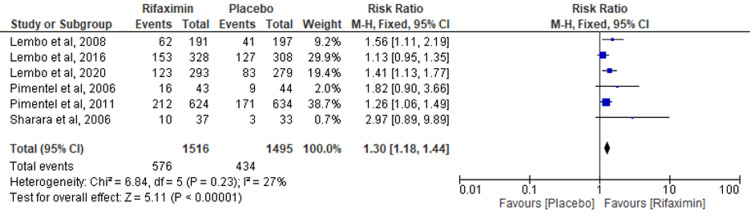
Overall symptom relief at the end of the follow-up period

Six RCTs analyzed the effectiveness of rifaximin versus placebo on overall symptom relief at the end of the follow-up period. It was observed that the results favored the rifaximin group over the placebo group (RR = 1.30; 95% CI: 1.18-1.44; p < 0.00001; I² = 27%) (Figure 3).

Comparison of Abdominal Pain

**Figure 4:**
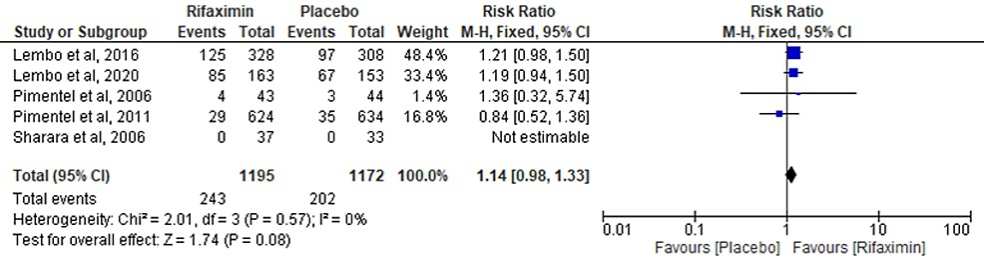
Comparison of abdominal pain

Five RCTs evaluated the outcome of abdominal pain. No significant differences were found between the groups of rifaximin and placebo (RR = 1.14; 95% CI: 0.98-1.33; p = 0.08; I² = 0%) (Figure 4).

Secondary outcomes

Comparison of Nausea

**Figure 5:**
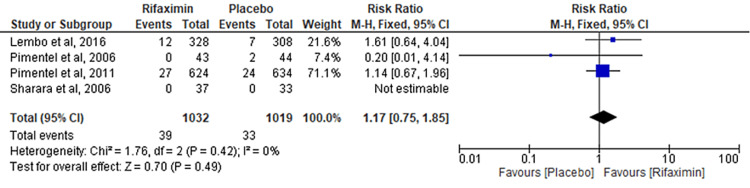
Comparison of nausea

Four RCTs were pooled to evaluate the comparison of nausea between the rifaximin and placebo groups. However, no significant results were observed between the two groups (RR = 1.17; 95% CI: 0.75-1.85; p = 0.49; I² = 0%) (Figure 5).

Comparison of Headache

**Figure 6:**
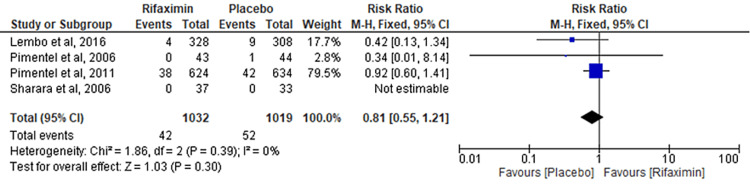
Comparison of headache

Four RCTs were pooled to evaluate the comparison of headaches between the rifaximin and placebo groups. No significant differences were observed between the two groups (RR = 0.81; 95% CI: 0.55-1.21; p = 0.30; I² = 0%) (Figure 6).

Comparison of Vomiting

**Figure 7:**
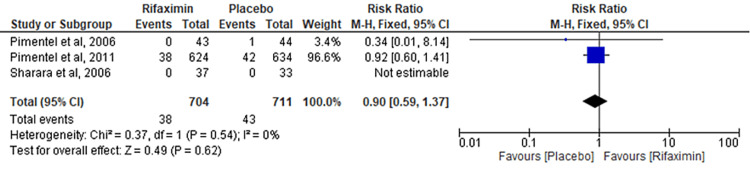
Comparison of vomiting

Three RCTs evaluated the comparison of vomiting between the rifaximin and placebo groups. No significant differences were found between the two groups (RR = 0.90; 95% CI: 0.59-1.37; p = 0.62; I² = 0%) (Figure 7).

Comparison of Abdominal Distension

**Figure 8:**
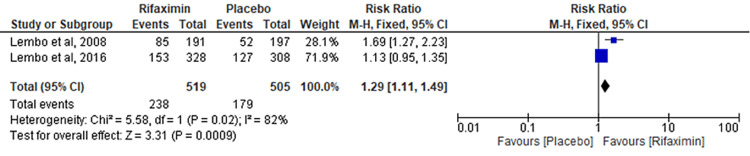
Comparison of abdominal distension

Two RCTs evaluated the difference between the rifaximin and placebo groups for the outcome of abdominal distension. Significant results were observed, favoring the rifaximin group over the placebo group (RR = 1.29; 95% CI: 1.11-1.49; p = 0.0009; I² = 82%) (Figure 8).

Comparison of Diarrhea

**Figure 9:**
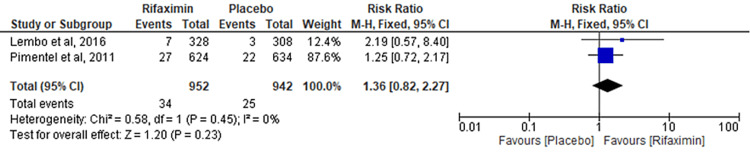
Comparison of diarrhea

Two RCTs evaluated the outcome of the comparison of diarrhea between the rifaximin and placebo groups. No significant difference was observed between the two groups (RR = 1.36; 95% CI: 0.82-2.27; p = 0.23; I² = 0%) (Figure 9).

Comparison of Sinusitis

**Figure 10:**
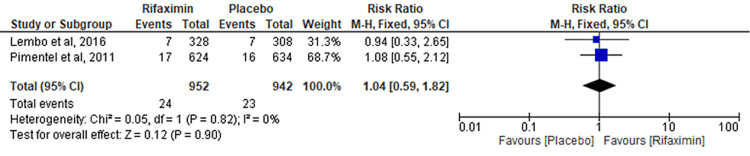
Comparison of sinusitis

Two RCTs evaluated the outcome of the comparison of sinusitis between the rifaximin and placebo groups. No significant difference was observed between the two groups (RR = 1.04; 95% CI: 0.59-1.82; p = 0.90; I² = 0%) (Figure 10).

Comparison of Bronchitis

**Figure 11:**
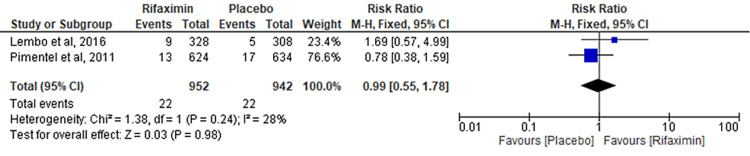
Comparison of bronchitis

Two RCTs evaluated the outcome of the comparison of bronchitis between the rifaximin and placebo groups. No significant difference was observed between the two groups (RR = 0.99; 95% CI: 0.55-1.78; p = 0.98; I² = 28%) (Figure 11).

Comparison of Upper Respiratory Tract Infection

**Figure 12:**
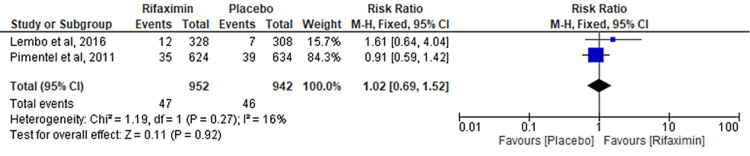
Comparison of upper respiratory tract infection

Two RCTs evaluated the outcome of the comparison of upper respiratory tract infections between the rifaximin and placebo groups. No significant difference was observed between the two groups (RR = 1.02; 95% CI: 0.69-1.52; p = 0.92; I² = 16%) (Figure 12).

Discussion

In our meta-analysis aimed at determining the impact of rifaximin on the symptoms of irritable bowel syndrome, we observed improved outcomes of symptom relief at the end of the treatment period, follow-up period, and abdominal distension in the rifaximin group compared to the placebo group. However, no significant difference was found between the two groups for the outcomes of abdominal pain, nausea, headache, vomiting, sinusitis, bronchitis, diarrhea, and upper respiratory tract infection.

The underlying causes of IBS are complex and involve a combination of factors. For instance, the balance of gut microorganisms affects both heightened sensitivity in the abdomen and immune system activation. In individuals with IBS, the composition of the gut microbiota is different from that of healthy individuals. One factor contributing to IBS development, particularly post-infectious IBS (PI-IBS), is acute gastroenteritis. Following such episodes, levels of the cytolethal-distending toxin (CdtB) protein may rise, and this increase has been linked to small intestinal bacterial overgrowth (SIBO) in animal models. Small intestinal bacterial overgrowth is more prevalent in IBS patients, especially those with certain characteristics like being female, older, or experiencing IBS-D [8, 10, 13].

Interestingly, despite systemic antibiotics being associated with IBS development, non-systemic antibiotic treatment is used to manage IBS, including IBS-D. Short, two-week courses of the antibiotic rifaximin have proven effective and well-tolerated for alleviating IBS symptoms in adults with IBS-D. While the precise mechanisms of rifaximin's effects aren't fully understood, indirect evidence suggests it has positive impacts on SIBO, reduction of mucosal inflammation, and stabilization of gut microorganisms. The available data indicate that rifaximin's action goes beyond being solely a gastrointestinal antibiotic. Further research is needed to address the gaps in our understanding of rifaximin's role in IBS. Both preclinical and clinical studies propose that rifaximin might normalize heightened abdominal sensitivity, decrease mucosal inflammation, influence the expression of immune modulators, and hinder gut permeability. It's crucial to conduct clinical studies incorporating surrogate markers to completely clarify how rifaximin modifies these causal factors associated with the underlying mechanisms of IBS [8, 10, 13].

Previous meta-analyses have evaluated the effectiveness of rifaximin in managing symptoms of irritable bowel syndrome [17-19]. Similar to the results of our meta-analysis (RR = 1.22; 95% CI: 1.12-1.33), Menees et al. (OR = 1.57; 95% CI: 1.22-2.01) and Li et al. (OR = 1.19; 95% CI: 1.8-1.32) observed a significant improvement in overall symptoms with rifaximin administration. However, unlike our analysis, Menees et al. [20] did not evaluate the data for follow-up after rifaximin initiation. Meanwhile, Li and colleagues also found a significant improvement in symptom relief after follow-up while using rifaximin (OR = 1.36; 95% CI: 1.18-1.58) [18]. Furthermore, similar to our analysis, Li et al. [18] found no significant association between rifaximin usage and the development of adverse events such as abdominal pain, nausea, vomiting, and headache. Ford et al. investigated the use of probiotics, antibiotics, synbiotics, and prebiotics for IBS [17]. Upon analysis, Ford found rifaximin to be more effective than placebo in non-constipated IBS patients, while no significant difference in the occurrence of adverse events could be noted. Black et al. found alosetron and ramosetron to be more effective than rifaximin for the treatment of IBS-D and IBS-M [20-21].

While Li et al. [18] evaluated the risk of developing adverse events, including abdominal pain, nausea, vomiting, and headache, our meta-analysis also investigated the risk of developing additional conditions such as abdominal distention, diarrhea, sinusitis, bronchitis, and upper respiratory tract infection due to rifaximin use. Moreover, our results revealed a significant risk of developing abdominal distention with rifaximin administration (RR = 1.29; 95% CI: 1.11-1.49). All of our primary and secondary outcomes demonstrated results with low heterogeneity, with the exception of the calculated RR for abdominal distention. No restrictions were placed on the type of IBS with which patients were diagnosed.

Limitations

Although our results demonstrate a significant risk of developing abdominal distention with rifaximin administration, they exhibit high heterogeneity (I² = 82%). Furthermore, due to the insufficient data available, we have not evaluated the effectiveness of rifaximin in terms of individual symptom relief or its impact on the development of adverse events beyond the follow-up period. Additionally, we have not explored the effects of rifaximin on specific populations, such as exclusively females or individuals within certain age groups, nor have we determined the optimal dosage of rifaximin for administration. We have also not evaluated data based on the type of diagnostic criteria (Rome I, II, or III) used in each RCT.

## Conclusions

The findings of our systematic review and meta-analysis strongly support the administration of rifaximin for treating irritable bowel syndrome due to its demonstrated safety and efficacy. Future randomized controlled trials and meta-analyses should be conducted to further evaluate the effectiveness of rifaximin therapy, aiming to enhance the quality of life for individuals with IBS.

## Figures and Tables

**Figure 13:**
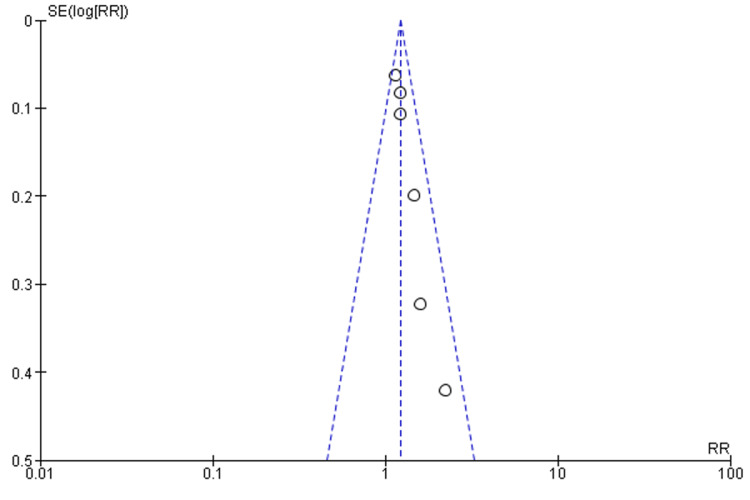
Primary endpoints, outcomes: 1.1 overall symptom relief at the end of the treatment period

**Figure 14:**
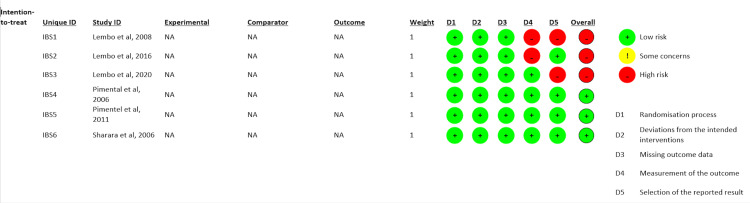
Quality assessment of the included studies.

## Appendices

Appendix A

**Table 2:** Search strategy

Database	Query	Search details	Results
PubMed	((((rifaximin) AND (irritable bowel syndrome)) OR (IBS)) OR (diarrhea)) AND (randomized controlled trial)	((("rifaximin"[Supplementary Concept] OR "rifaximin"[All Fields] OR "rifaximin"[MeSH Terms] OR "rifaximine"[All Fields]) AND ("irritable bowel syndrome"[MeSH Terms] OR ("irritable"[All Fields] AND "bowel"[All Fields] AND "syndrome"[All Fields]) OR "irritable bowel syndrome"[All Fields])) OR "IBS"[All Fields] OR ("diarrhea"[MeSH Terms] OR "diarrhea"[All Fields] OR "diarrheas"[All Fields] OR "diarrhoea"[All Fields] OR "diarrhoeas"[All Fields])) AND ("randomized controlled trial"[Publication Type] OR "randomized controlled trials as topic"[MeSH Terms] OR "randomized controlled trial"[All Fields] OR "randomised controlled trial"[All Fields])	10, 572
Web of Science			
Embase			
The Cochrane Library			
Scopus			
Google Scholar			

Appendix B

Appendix C
